# Wnt–*β*-catenin–Tcf-4 signalling-modulated invasiveness is dependent on osteopontin expression in breast cancer

**DOI:** 10.1038/bjc.2011.269

**Published:** 2011-07-19

**Authors:** A Ravindranath, H-F Yuen, K-K Chan, C Grills, D A Fennell, T R Lappin, M El-Tanani

**Affiliations:** 1 Centre for Cancer Research and Cell Biology (CCRCB), Queen's University Belfast, Belfast BT9 7BL, UK

**Keywords:** Tcf-4, osteopontin, Wnt signalling, breast cancer, survival

## Abstract

**Background:**

We have previously demonstrated that Tcf-4 regulates osteopontin (OPN) in rat breast epithelial cells, Rama37. In this report, we have examined the importance of this regulation in human breast cancer.

**Methods:**

The regulatory roles of Tcf-4 on cell invasion and OPN expression were investigated. The mRNA expression of Tcf-4 and OPN, and survival of breast cancer patients were correlated.

**Results:**

Tcf-4 enhanced cell invasion in both MCF10AT and MDA MB 231 breast cancer cells by transcriptionally activating OPN expression. Osteopontin was activated by Wnt signalling in MDA MB 231 cells. Paradoxical results on Tcf-4-regulated OPN expression in MCF10AT (activation) and Rama37 (repression) cells were shown to be a result of differential Wnt signalling competency in MCF10AT and Rama37 cells. High levels of OPN and Tcf-4 mRNA expression were significantly associated with survival in breast cancer patients. Most importantly, Tcf-4-positive patients had a poorer prognosis when OPN was overexpressed, while OPN-negative patients had a better prognosis when Tcf-4 was overexpressed.

**Conclusion:**

Our results suggest that Tcf-4 can act as a repressor or activator of breast cancer progression by regulating OPN expression in a Wnt-dependent manner and that Tcf-4 and OPN together may be a novel prognostic indicator for breast cancer progression.

Wnt signalling has an important role in breast cancer initiation and progression (Smalley and Dale, 1999; Farago *et al*, 2005; Ayyanan *et al*, 2006; Zhang *et al*, 2010). Although there is no evidence for specific mutations, the accumulation of *β*-catenin, which mediates Wnt signalling-induced cancer progression (Michaelson and Leder, 2001), suggests that this signalling pathway is aberrantly activated in breast cancer (Brennan and Brown, 2004). In addition, inhibitors of Wnt signalling, such as WIF-1, are aberrantly downregulated in breast cancer (Wissmann *et al*, 2003; Ai *et al*, 2006; Suzuki *et al*, 2008). Wnt signalling also promotes metastatic progression (Yook *et al*, 2006; Matsuda *et al*, 2009), self-renewal (DiMeo *et al*, 2009) and radiation resistance (Woodward *et al*, 2007) in breast cancer. Moreover, in human breast cancer specimens, Wnt activation is associated with poor prognosis (Veeck *et al*, 2006; Khramtsov *et al*, 2010).

Tcf-4 promotes colon cancer initiation and progression (Roose *et al*, 1999; Takahashi *et al*, 2002; van de Wetering *et al*, 2002; Pannequin *et al*, 2007). However, in breast cancer, paradoxical results for the role of Tcf-4 on cancer progression have been reported. Tcf-4 binds to *β*-catenin to transactivate Wnt target genes (Graham *et al*, 2001). However, in the absence of *β*-catenin, Tcf-4 becomes a repressor of transcription of those target genes *via* binding to corepressors such as Groucho (Hoverter and Waterman, 2008). This characteristic may explain the apparently inconsistent findings that Tcf-4 can either promote or repress breast cancer progression in different cellular contexts. We have previously shown that Tcf-4 binds to a metastasis-inducing DNA sequence (El-Tanani *et al*, 2001c). This results in a reduction in the amount of Tcf-4 available to regulate the expression of endogenous genes, leading to a decrease in metastatic development (El-Tanani *et al*, 2001a). Others have also shown that Tcf-4 has a tumour suppressor function in breast cancer (Shulewitz *et al*, 2006; Beildeck *et al*, 2009). In contrast, overexpression of Tcf-4 in combination with *β*-catenin in breast cancer cells led to increased expression of monocyte chemotactic protein-1, which promotes cancer progression (Mestdagt *et al*, 2006).

The reasons for the paradoxical results obtained on the role of Tcf-4 in breast cancer progression are not clear. As we have previously shown that osteopontin (OPN) is a downstream target of Tcf-4 in a rat breast epithelial cell line Rama37 (El-Tanani *et al*, 2001a), the aim of the present study was to investigate how the Tcf-4/OPN link is regulated in human breast cancer and modulates human breast cancer progression.

## Materials and methods

### Cell culture

MCF10AT cells (Karmonas Cancer Institute, Detroit, MI, USA) were grown in DMEM/F12 (1 : 1) (Invitrogen, Paisley, UK) supplemented with 5% (v/v) horse serum (Invitrogen), 10 *μ*g ml^−1^ (w/v) insulin (Sigma-Aldrich, Gillingham, UK), 0.5 *μ*g ml^−1^ (w/v) hydrocortisone (Sigma-Aldrich), 20 *μ*g ml^−1^ (w/v) epidermal growth factor (Sigma-Aldrich) and 100 ng ml^−1^ (w/v) cholera enterotoxin (Sigma-Aldrich). MDA MB 231 (a gift from Professor Robert A Weinberg, Whitehead Institute, Boston, MA, USA) and Rama37 cells were cultured in DMEM with high glucose (Invitrogen) supplemented with 10% (v/v) fetal bovine serum (FBS, Invitrogen). Cells were maintained in medium supplemented with 1 × penicillin and streptomycin (Invitrogen). Transfection was achieved using GeneJuice according to the manufacturer's instructions (Novagen, Darmstadt, Germany). For stable MCF10AT pcDNA6 and MCF10AT pcDNA6-Tcf-4 cells, the transfectants were selected 24 h post-transfection in medium containing 4 *μ*g ml^−1^ Blasticidin S HCl (Invitrogen). Transfectant colonies were isolated after 2 weeks, when all the cells in the untransfected control were dead, and were pooled to generate stable transfectants for biological assay. Transient transfection of pCMV7.1-Flag-OPN and pcDNA3.1-DKK1 (a gift from Professor KW Chan; Yuen *et al*, 2008) was also performed using GeneJuice. Cells were harvested 48 h post-transfection for western blot, promoter analyses and invasion assay. The Top/Fop glow reporter system was obtained from Upstate (Millipore, Billerica, MA, USA).

### RNA interference for Tcf-4 and *β*-catenin in MDA MB 231 cells

MDA MB 231 cells were transfected with scrambled siRNA (AM4611, Ambion, Austin, TX, USA), siRNA duplexes directed against Tcf-4 (AM16704 ID #116411, Ambion) or *β*-catenin (M-003482, Dharmacon, Chicago, IL, USA) using siPORT NeoFX (Ambion) transfection reagent. Cells were harvested 48 h post-transfection for western blot and promoter analyses, and invasion assay.

### Western blotting

Total protein lysate was prepared by using RIPA buffer. Nuclear and cytoplasmic protein was prepared by using NE-PER nuclear extraction kit according to the manufacturer's instructions (Pierce, Thermo Fisher Scientific Inc., Rockford, IL, USA). Western blotting was performed as previously described (El-Tanani *et al*, 2001a) at the following dilution of antibodies: 1 : 250 for Tcf-4 (05-511, Millipore), 1 : 150 for OPN (sc73631, Santa Cruz Biotechnology Inc., Santa Cruz, CA, USA), 1 : 2500 for *β*-catenin (610154, BD Transduction Laboratories, Franklin Lakes, NJ, USA), 1 : 2000 for Dkk1 (AF1096, R&D Systems Inc., Minneapolis, MN, USA) and 1 : 5000 for *β*-actin (A5441, Sigma-Aldrich) were used.

### Real-time PCR

Quantitative PCR was performed using the ABI PRISM 7700 (Applied Biosystems, Foster City, CA, USA) according to the manufacturer's instructions. The Taqman probe sets for Tcf-4 (assay id Hs01009038_m1), OPN (assay id Hs00959009_m1) and 18s RNA (assay id 319913E-0702028) were used. Gene expression was normalised to 18s RNA.

### *In silico* analysis of human OPN regulatory sequence

A 3.5-kb region of human OPN gene upstream of the Transcriptional Start Site (TSS) was analysed for the consensus transcription factor binding region using MatInspector Tool from Genomatix Software GmbH (Munich, Germany), which utilises a comprehensive transcription factor knowledge base known as MatBase.

### Luciferase reporter assay

The luciferase reporter assay was performed as previously described (El-Tanani *et al*, 2001b).

### Chromatin immunoprecipitation assay

Chromatin immunoprecipitation (ChIP) was performed as previously described (Yuen *et al*, 2008). Mouse IgG (Santa Cruz Biotechnology) was used as a control. Touch-down PCR was performed using the precipitated DNA as template and cycling conditions as follows: 10 cycles for denaturation at 98 °C for 10 s, annealing ramping from 70 to 60 °C for 10 s and extension at 72 °C for 5 s; 30 cycles for denaturation at 98 °C for 10 s, annealing at 66.2 °C for 10 s and extension at 72 °C for 5 s, followed by final extension at 72 °C for 5 min. The primers used to amplify the DNA region containing the Tcf/Lef binding site within the human OPN promoter were forward: 5′-GCCTAAGGCAACAACAGAGC-3′ reverse: 5′-TCCAGCGGGATAGAACACTC-3′.

### Immunofluorescence staining

Immunofluorescence staining was performed as previously described (Yuen *et al*, 2011).

### Matrigel invasion assay

Cell invasion assay was performed as previously described (Shi *et al*, 2010).

### Analysis of breast cancer patient microarray data

A total of three breast cancer data sets (GSE1456, GSE3494 and GSE1379), consisting of 455 patients, for whom corresponding microarray and survival data are available in the Gene Expression Omnibus (GEO) database, were included in this study. The data sets were pre-processed as previously described (Yuen *et al*, 2011) using R and Bioconductor for normalisation. For studies with raw microarray data that had >20% missing values across all arrays per study were excluded, otherwise missing values were replaced by median values per gene. Normalisation was conducted using the RMA algorithm in Bioconductor. The median and higher quartile values for OPN and Tcf-4 were used as cutoff points to differentiate between high and low levels of expression for survival analysis.

### Statistical analysis

The Student's *t*-test was used to compare the statistical significance between the two sets of *in vitro* data. ^*^, ^**^ and ^***^ represent *P*<0.05, *P*<0.01 and *P*<0.001, respectively. Correlations between mRNA expression levels of OPN and Tcf-4 with overall survival were investigated by Kaplan–Meier analysis and compared by Log-rank test. *P*<0.05 was considered statistically significant.

## Results

### Tcf-4 promotes invasion in human breast cancer cell lines MCF10AT and MDA MB 231

**Figure 1:**
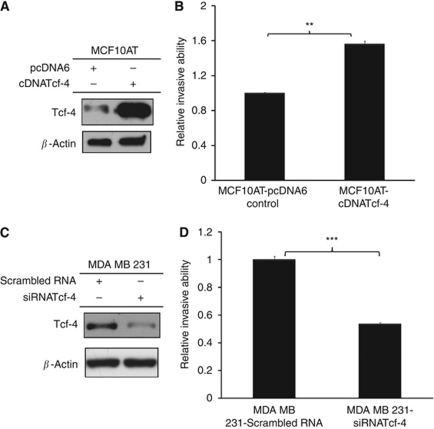
Tcf-4 promotes cell invasion in both MCF10AT and MDA MB 231 human breast cancer cells. (**A**) Western blotting showing overexpression of Tcf-4 in MCF10AT cells. (**B**) Overexpression of Tcf-4 in MCF10AT cells resulted in an increase in cell invasion through Matrigel-coated chamber. (**C**) Western blotting showing knockdown of Tcf-4 in MDA MB 231 cells. (**D**) Reduction in Tcf-4 expression in MDA MB 231 cells resulted in a decrease in cell invasion through Matrigel-coated chamber. Key: ^**^ and ^***^ represent *P*<0.01 and *P*<0.001, respectively.

We initiated our analysis by generating stable Tcf-4 overexpressing MCF10AT cells (Dawson *et al*, 1996). Overexpression of Tcf-4 resulted in a six-fold increase in Tcf-4 protein levels (Figure 1A). Although MCF10AT cells are tumourigenic, they are non-metastatic and unable to invade and migrate to distant sites (Miller *et al*, 1993). *In vitro* invasion assays were performed to investigate the effect of overexpressing Tcf-4 on the invasiveness of MCF10AT cells. Tcf-4 overexpressing MCF10AT cells showed a 56% higher invasive ability than the vector control cells as determined by the relative invasion rate of the transfected cells through Matrigel-coated Boyden chambers (Figure 1B). siRNA-mediated knockdown of Tcf-4 in MDA MB 231 cells resulted in an 80% reduction in endogenous Tcf-4 protein levels (Figure 1C) and a significant 47% decrease in the invasive ability through Matrigel-coated Boyden chambers compared with scrambled siRNA control cells (Figure 1D). These results highlight the important role of Tcf-4 in promoting invasion of MCF10AT and MDA MB 231 breast cancer cells.

### Tcf-4 transactivates OPN in human breast cancer cell lines MCF10AT and MDA MB 231

**Figure 2:**
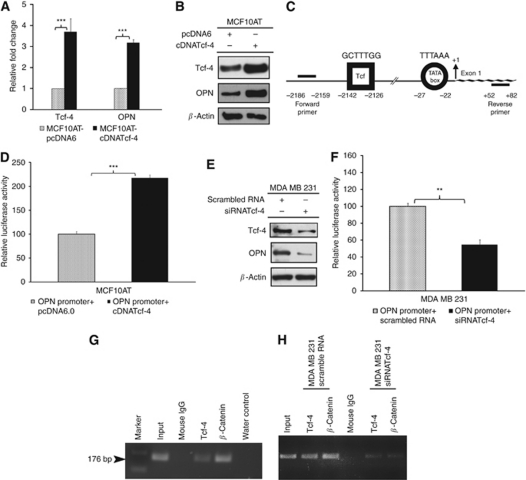
Tcf-4 transactivates OPN in both MCF10AT and MDA MB 231 human breast cancer cells. (**A**) Tcf-4 overexpression in MCF10AT resulted in an increase in both Tcf-4 and OPN mRNA expression. (**B**) Tcf-4 overexpression in MCF10AT resulted in an increase in OPN protein expression. (**C**) Schematic diagram of human OPN promoter showing a Tcf-4 binding site predicted by MatInspector Tool from Genomatix Software GmbH. (**D**) Tcf-4 overexpression resulted in an increase in the human OPN promoter activity in MCF10AT cells. (**E**) Knockdown of Tcf-4 in MDA MB 231 cells resulted in a decrease in expression of OPN protein. (**F**) Knockdown of Tcf-4 in MDA MB 231 cells resulted in a decrease in human OPN promoter activity. (**G**) ChIP analysis showing binding of Tcf-4 and *β*-catenin to an OPN promoter region containing the Tcf-4 binding site. (**H**) ChIP analysis showing the amount of OPN promoter precipitated by anti-Tcf-4 or anti-*β*-catenin antibodies was reduced in MDA MB 231 cells transfected with siRNA targeting Tcf-4 than that in MDA MB 231 cells transfected with scrambled siRNA. Key: ^**^ and ^***^ represent *P*<0.01 and *P*<0.001, respectively.

The role of Tcf-4 in regulating OPN expression was investigated. In Tcf-4 overexpressing MCF10AT cells, Tcf-4 mRNA was upregulated 3.6-fold and endogenous OPN protein level was increased 3.2-fold, both significantly higher than the vector control cells (Figure 2A). The increase in the mRNA of Tcf-4 and OPN was in concordance with the upregulation of their protein levels as shown in western blot analysis (Figure 2B). To investigate whether Tcf-4 upregulates OPN through modulating OPN promoter activity as it does in Rama37 cells (El-Tanani *et al*, 2001a), *in silico* analysis of the 3.5-kb region of human OPN gene upstream of the TSS, using MatInspector Tool (Quandt *et al*, 1995) from Genomatix Software GmbH, was performed. It revealed the presence of a Tcf/Lef binding site (GAACG**CTTTG**GTTCTCT) at −2142 bp to −2126 bp upstream of TSS (Figure 2C). This result prompted us to investigate whether OPN is transcriptionally regulated by Tcf-4 in MCF10AT cells. We isolated a 2.3-kb human OPN promoter region containing the Tcf binding site using touch-down PCR from Human chromosome 4 clone (RZPDB737F091018D, imaGenes, Berlin, Germany) and cloned it into the luciferase pGL3 basic reporter plasmid. Transient overexpression of Tcf-4 led to a two-fold increase in the human OPN promoter activity (Figure 2D), suggesting that Tcf-4 transactivates the human OPN promoter.

To investigate whether the endogenous Tcf-4 is important for OPN expression, OPN protein expression and promoter activity were investigated in MDA MB 231 cells with or without Tcf-4 knockdown. siRNA-mediated Tcf-4 knockdown in MDA MB 231 cells led to a 54% reduction in endogenous OPN protein levels as compared with the scrambled siRNA control cells (Figure 2E) and a concomitant 45% decrease in the activity of the OPN promoter (Figure 2F).

To determine whether the potential Tcf/Lef binding site on the endogenous OPN promoter is occupied by endogenous Tcf-4 and *β*-catenin proteins, a ChIP assay was performed using human breast cancer cell line MDA MB 231, which expresses high levels of Tcf-4, *β*-catenin and OPN. Immunoprecipitation using either Tcf-4 or *β*-catenin antibody specifically enriched the human OPN promoter DNA fragment containing the putative Tcf/Lef binding site (Figure 2G). Thus, the ChIP assay shows that endogenous Tcf-4 and *β*-catenin proteins bind to the Tcf/Lef binding site in the human OPN promoter. To confirm the binding of *β*-catenin to the human OPN promoter is mediated by Tcf-4, the amount of human OPN promoter bound by Tcf-4 and *β*-catenin was compared between MDA MB 231 cells transfected with scramble siRNA or siRNA targeting Tcf-4. As shown in Figure 2H, by ChIP assay, we found that the amount of human OPN precipitated by both Tcf-4 and *β*-catenin antibodies was reduced in MDA MB 231 cells transfected with siRNA targeting Tcf-4 compared with MDA MB 231 cells transfected with scramble siRNA. This result suggests that the binding of *β*-catenin to human OPN promoter is mediated by Tcf-4.

Taken together, our results suggest that the Tcf-4/*β*-catenin complex may occupy the Tcf/Lef binding site in the human OPN promoter in MDA MB 231 cells and transactivate the endogenous OPN promoter, thereby enhancing the mRNA and protein expression of OPN.

### OPN is a downstream target gene of Tcf-4-enhanced cell invasion in MCF10AT and MDA MB 231 cells

**Figure 3:**
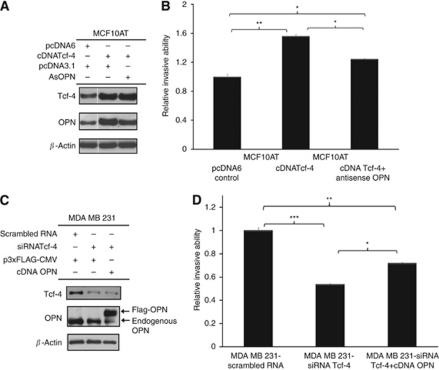
OPN is a downstream target of Tcf-4-enhanced cell invasion in MCF10AT and MDA MB 231 cells. (**A**) Western blotting showing that Tcf-4 overexpression resulted in an increase in OPN protein, which was knocked down by antisense OPN in MCF10AT cells. (**B**) Tcf-4-enhanced cell invasion in MCF10AT cells was significantly reduced by knocking down OPN expression. (**C**) Transfection of Tcf-4 siRNA resulted in a decrease in the expression of both endogenous Tcf-4 and OPN protein levels and transfection of Flag-OPN resulted in overexpression of exogenous Flag-OPN protein in MDA MB 231 cells. (**D**) Tcf-4 knockdown-mediated decrease in cell invasion in MDA MB 231 cells was partially reversed by overexpression of exogenous Flag-OPN. Key: ^*^, ^**^ and ^***^ represent *P*<0.05, *P*<0.01 and *P*<0.001, respectively.

The enhanced OPN expression was stably knocked down using an antisense OPN construct in Tcf-4 overexpressing MCF10AT cells (Figure 3A). Osteopontin knockdown using the antisense OPN construct resulted in a significant 20% reduction of the Tcf-4-mediated increase in cell invasion through Matrigel in MCF10AT cells (Figure 3B). On the other hand, OPN overexpression in MDA MB 231-siRNATcf-4 cells (Figure 3C) resulted in a significant 35% increase of the siRNATcf-4-mediated reduction in cell invasion of MDA MB 231 cells (Figure 3D). These results suggest that Tcf-4-regulated OPN expression may have an important role in Tcf-4-mediated promotion in cell invasion in both MCF10AT and MDA MB 231 breast cancer cells.

### OPN is positively regulated by Wnt signalling

**Figure 4:**
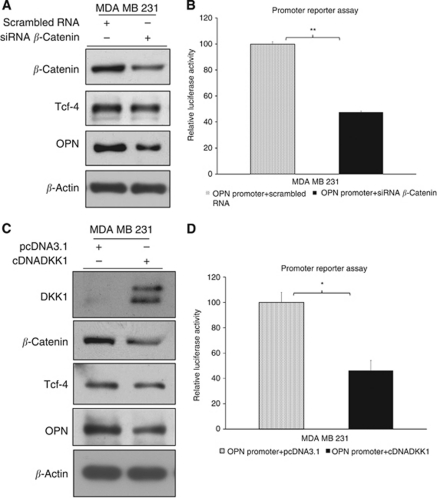
OPN is positively regulated by Wnt signalling in MDA MB 231 cells. Knockdown of *β*-catenin resulted in (**A**) a decrease in *β*-catenin and OPN protein expression and (**B**) a reduction in human OPN promoter activity. Overexpression of DKK-1 resulted in (**C**) an increase in DKK-1, a decrease in both *β*-catenin and OPN protein expression and (**D**) a reduction in human OPN promoter activity. Key: ^*^ and ^**^ represent *P*<0.05 and *P*<0.01, respectively.

The invasive breast cancer cell line MDA MB 231 expresses high levels of OPN (McAllister *et al*, 2008) and has aberrantly activated Wnt signalling (Matsuda *et al*, 2009). We, therefore, investigated whether the high level expression of OPN in MDA MB 231 cells is caused by the activated Wnt signalling. Transient knockdown using siRNA targeting *β*-catenin in MDA MB 231 cells decreased *β*-catenin protein by 51% and endogenous OPN protein expression by 55% (Figure 4A). A 46% reduction in the activity of the human OPN promoter reporter construct upon *β*-catenin knockdown as compared with scrambled siRNA control cells was also observed (Figure 4B). On the other hand, overexpression of Dickkopf-related protein 1 (DKK1), an inhibitor of Wnt signalling, in MDA MB 231 cells decreased endogenous *β*-catenin protein levels, due to the decreased stability of *β*-catenin upon reduction of Wnt activity, compared with empty vector transfected cells. DKK1-mediated Wnt signalling inactivation in MDA MB 231 cells resulted in a 30% reduction in endogenous OPN protein (Figure 4C) as well as a 54% reduction in the activity of the human OPN promoter reporter construct (Figure 4D). These results suggest that aberrant activation of Wnt signalling in MDA MB 231 cells may account for the high level expression of OPN.

### Tcf-4-induced OPN may be dependent on the competency of Wnt signalling

**Figure 5:**
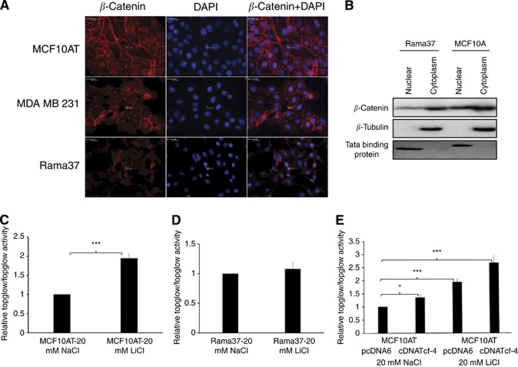
Wnt signalling activity in human MCF10AT and rat Rama37 breast epithelial cells. (**A**) *β*-Catenin was highly expressed in both nuclear and cytoplasmic regions in MCF10AT (upper panel) and MDA MB 231 (middle panel) cells and was lower in Rama37 cells (lower panel). (**B**) Nuclear/cytoplasmic fractionation showing that the nuclear to cytoplasmic *β*-catenin expression ratio was higher in MCF10A cells than in Rama37 cells. (**C**) Treatment of LiCl resulted in an increase in Top/Fop-activity ratio in MCF10AT cells. (**D**) Treatment of LiCl did not significantly change Top/Fop-activity ratio in Rama37 cells. (**E**) Overexpression of Tcf-4 resulted in an increase in Top/Fop-activity ratio in the presence and absence of LiCl-mediated stimulation. Key: ^*^ and ^***^ represent *P*<0.05 and *P*<0.001, respectively.

Previous reports indicate that Tcf-4 works as an activator in the presence of Wnt signalling through binding to the promoter in a complex with *β*-catenin whereas it works as a repressor in the absence of Wnt (Bienz, 1998). This may explain the paradoxical results obtained from Rama37 (where Tcf-4 suppresses OPN) and MCF10AT cells (where Tcf-4 transactivates OPN). Therefore, we investigated whether the two cell lines have different patterns of Wnt signalling. Immunofluorescence staining showed high levels of both membrane and nuclear expression of *β*-catenin in MCF10AT cells (Figure 5A, top panel). A similar staining pattern was also found in MDA MB 231 cells, which are Wnt positive (Figure 5A, middle panel). In contrast, the *β*-catenin expression was lower in both subcellular locations in Rama37 cells (Figure 5A, bottom panel). The expression levels of *β*-catenin in nucleus and cytoplasm of Rama37 and MCF10A were compared by western blot. The nuclear to cytoplasmic ratio of *β*-catenin in MCF10A cells was higher than that in Rama37 cells, further suggesting that the basal Wnt activity may be higher in MCF10A cells than in Rama37 cells (Figure 1B). To investigate the integrity of Wnt signalling in MCF10AT and Rama37 cells, we tested the Top/Fop glow reporter activity in Rama37 and MCF10AT cells treated with lithium chloride (LiCl), which inhibits GSK-3*β* and thereby increases the stability of *β*-catenin (Stambolic *et al*, 1996), or sodium chloride (NaCl) as a control. Lithium chloride treatment showed a two-fold increase in Tcf/*β*-catenin transcriptional activity (Figure 5C) in MCF10AT cells compared with cells treated with NaCl, suggesting that stabilisation of *β*-catenin enhances the Wnt-dependent transcriptional activity, whereas LiCl treatment did not show any significant change in the Tcf/*β*-catenin transcriptional activity in Rama37 cells (Figure 5D). This result is in line with our previous data, which showed that overexpression of Tcf-4 results in downregulation of its downstream target OPN (El-Tanani *et al*, 2001a). Indeed, Tcf-4 overexpression alone in MCF10AT cells transactivated the Wnt signalling reporter construct (Figure 5E). Taken together, our results show that Tcf-4 functions as an activator in MCF10AT cells but not in Rama37 cells because *β*-catenin is more available in MCF10AT than in Rama37 cells and Wnt activity can be triggered in MCF10AT cells upon treatment with Wnt activating chemical, LiCl, but not in Rama37 cells. These results suggest that the activity of Wnt and availability of *β*-catenin may be important in determining whether Tcf-4 is an activator or repressor of OPN, thereby the invasion potential.

### The prognostic significance of Tcf-4 and OPN mRNA expression in human breast cancers

**Figure 6:**
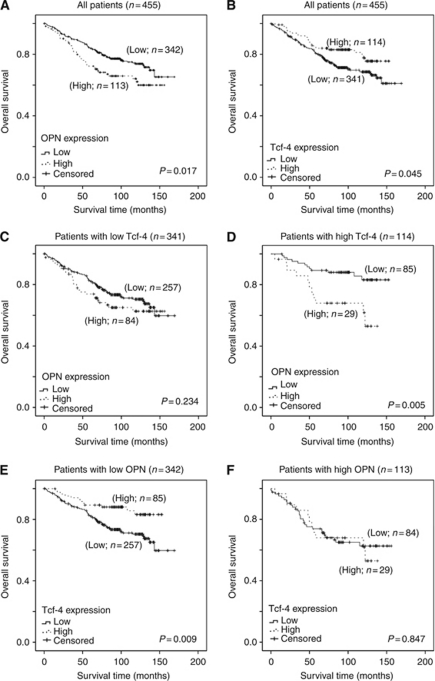
The prognostic significance of OPN and Tcf-4 mRNA expression (cutoff point at higher quartile) in breast cancer patients. (**A**) High levels of OPN mRNA were significantly associated with a shorter survival time of breast cancer patients. (**B**) Low levels of Tcf-4 mRNA were significantly associated with a shorter survival time of breast cancer patients. (**C**) In patients expressing low levels of Tcf-4 mRNA, mRNA expression of OPN was not significantly correlated with survival. (**D**) In patients expressing high levels of Tcf-4 mRNA, high levels expression of OPN mRNA were correlated with a shorter survival time. (**E**) In patients expressing low levels of OPN mRNA, low levels expression of Tcf-4 mRNA were correlated with a shorter survival time. (**F**) In patients expressing high levels of OPN mRNA, mRNA expression of Tcf-4 was not significantly correlated with survival.

mRNA expression levels of Tcf-4 and OPN, and the survival data of patients were retrieved from three data sets (GSE 1397, GSE 1456 and GSE 3494) available from the GEO database and the three data sets were combined as one. The higher quartile of the mRNA expression levels was used as a cutoff point in the present study for Kaplan–Meier survival analysis. High levels of OPN mRNA were correlated with a shorter survival time in the combined data set (0.017; Figure 6A), consistent with our previous study (Rudland *et al*, 2002). In contrast, low levels of Tcf-4 mRNA were correlated with a shorter survival time in the combined data set (*P*=0.045; Figure 6B). These results are in line with our previous study and others showing that OPN may promote, while Tcf-4 may inhibit, breast cancer progression (El-Tanani *et al*, 2001a; Shulewitz *et al*, 2006; Beildeck *et al*, 2009). A correlation between OPN and patient survival was only observed in patients expressing high levels of Tcf-4. Thus, OPN mRNA levels were not significantly correlated with survival in patients expressing low levels of Tcf-4 (*P*=0.234; Figure 6C). In contrast, high levels of OPN mRNA were significantly correlated with a shorter survival time in patients expressing high levels of Tcf-4 (*P*=0.005; Figure 6D). In addition, we revealed that the correlation between Tcf-4 and survival was only significant in patients expressing low levels of OPN (*P*=0.009; Figure 6E) but not those expressing high levels (*P*=0.847; Figure 6F). To avoid bias in selection of cutoff point, we also re-analysed the results using median, instead of higher quartile, mRNA expression level as the cutoff point for Kaplan–Meier analysis. Similar results were obtained for both cutoff points (Supplementary Figure 1). Taken together, our results indicate that the functional role of Tcf-4 in breast cancer progression may be determined by Wnt-dependent OPN regulation and expression, and that OPN and Tcf-4 may be used in combination as a novel prognostic indicator.

## Discussion

In the present study, we have shown that Tcf-4 promoted cell invasion through transcriptional activation of OPN, a metastasis-promoting protein, in MCF10AT and MDA MB 231 cells. We also found that OPN was positively regulated by Wnt signalling in invasive breast cancer cell line MDA MB 231 and that Tcf-4 activation of OPN transcription might be dependent on Wnt signalling activity. Most importantly, Tcf-4 mRNA expression was correlated with better survival, while OPN mRNA expression was correlated with poorer survival in breast cancer patients. Their prognostic significance was also found to be dependent on the expression levels of the others.

OPN mRNA only predicted a shorter survival time in patients expressing high levels of Tcf-4, implying that if a high level of Tcf-4 is accompanied by a high level of OPN, the patients have a poorer prognosis. The scenario observed in MCF10AT cells may reflect the clinical condition of these patients. Thus, Tcf-4 overexpression in a Wnt-positive background upregulates OPN, resulting in high levels of both Tcf-4 and OPN, thereby promoting cell invasion and cancer progression. On the other hand, in patients expressing low levels of OPN, Tcf-4 predicted a better survival, implying that in a low OPN background, Tcf-4 may act as a tumour suppressor in breast cancer patients. The scenario observed in Rama37 cells (El-Tanani *et al*, 2001a) may be mirrored by what is occurring in these patients. Thus, Tcf-4 overexpression in a Wnt-negative background fails to upregulate OPN, resulting in a high level of Tcf-4 but a low level of OPN, thereby suppressing cell invasion and cancer progression.

The results obtained in the patient data set correlated with the seemingly paradoxical *in vitro* results obtained in MCF10AT (current study) and in Rama37 (El-Tanani *et al*, 2001a) cells. This observation is interesting because OPN mRNA expression levels can differentiate aggressive or indolent breast cancers only in patients expressing high levels of Tcf-4 mRNA expression, suggesting that the tumour suppressor function of Tcf-4 may only be expressed in OPN-low background (in the case of Rama37 cells where Tcf-4 suppresses OPN) but not in OPN-high background (in the case of MCF10AT cells where Tcf-4 activates OPN). Our results obtained from human data, therefore, correlate with our *in vitro* data, suggesting that Wnt-dependent OPN regulation/expression may have an important role in Tcf-4-mediated alteration of cell invasion. However, further investigations are required to substantiate this hypothesis. Immunohistochemical staining should be performed to study the protein expression, instead of mRNA expression, of Tcf-4 and OPN in a large human breast cancer patient cohort to confirm the correlations between OPN, Tcf-4 and patient survival time. Moreover, Wnt signalling activity should also be examined in the tumour specimens to investigate the effect of Wnt activity on the expression and prognostic significance of Tcf-4 and OPN.

*β*-Catenin knockdown and DKK-1 overexpression in MDA MB 231, where the Wnt signalling activity is reduced, also resulted in slight reduction in Tcf-4 protein level (15% reduction in *β*-catenin knockdown and 29% reduction in DKK-1 overexpression). Tcf-4 has been shown to be regulated transcriptionally by Wnt signalling but how this autoregulation of Tcf-4 affects OPN expression requires further analysis.

The evidence so far indicates that OPN functions as a nodal point in multiple signalling networks that lead to malignant transformation. This underscores the need for elucidating the OPN regulatory networks, which may ultimately help in the identification of novel targets for therapies directed against OPN-positive cancers. Osteopontin is an important factor in cancer progression and metastasis (Coppola *et al*, 2004). We have shown previously (Rudland *et al*, 2002) and in the present study that breast cancer patients with OPN-positive tumours have a significantly shorter survival time. In the present study, we also show that OPN is a target of Wnt signalling in a human breast cancer cell line, MDA MB 231. Our results may stimulate others to investigate the possibility of using Wnt inhibitors as therapeutic approaches for Wnt/OPN double-positive breast cancers.

The effect of OPN on Tcf-4-mediated invasive progression is partial. This highlights the role of other Wnt target genes downstream of Tcf-4 in Tcf-4-modulated cell invasion in breast cancer cells. The Tcf-4/*β*-catenin signalling pathway has an important role in expression of urokinase plasminogen activator (Mann *et al*, 1999), metalloproteinase 1 (MMP-1; Takahashi *et al*, 2002) as well as MMP-7 (Brabletz *et al*, 1999), which have an important function in breast cancer metastatic progression. Investigation of the expression levels and their roles in Tcf-4 overexpressing MCF10AT or Tcf-4 knockdown MDA MB 231 cells may provide new mechanistic insights, in addition to OPN, on Wnt-mediated breast cancer progression.

In conclusion, our results suggest that Tcf-4-regulated OPN expression and cell invasion may be dependent on Wnt signalling activity and that Tcf-4 and OPN, when considered together, may be a novel prognostic indicator in breast cancer.

## References

[bib1] Ai L, Tao Q, Zhong S, Fields CR, Kim WJ, Lee MW, Cui Y, Brown KD, Robertson KD (2006) Inactivation of Wnt inhibitory factor-1 (WIF1) expression by epigenetic silencing is a common event in breast cancer. Carcinogenesis 27(7): 1341–1348 16501252 10.1093/carcin/bgi379

[bib2] Ayyanan A, Civenni G, Ciarloni L, Morel C, Mueller N, Lefort K, Mandinova A, Raffoul W, Fiche M, Dotto GP, Brisken C (2006) Increased Wnt signaling triggers oncogenic conversion of human breast epithelial cells by a Notch-dependent mechanism. Proc Natl Acad Sci USA 103(10): 3799–3804 16501043 10.1073/pnas.0600065103 PMC1450156

[bib3] Beildeck ME, Islam M, Shah S, Welsh J, Byers SW (2009) Control of TCF-4 expression by VDR and vitamin D in the mouse mammary gland and colorectal cancer cell lines. PLoS One 4(11): e7872 19924301 10.1371/journal.pone.0007872 PMC2774944

[bib4] Bienz M (1998) TCF: transcriptional activator or repressor? Curr Opin Cell Biol 10(3): 366–372 9640538 10.1016/s0955-0674(98)80013-6

[bib5] Brabletz T, Jung A, Dag S, Hlubek F, Kirchner T (1999) Beta-catenin regulates the expression of the matrix metalloproteinase-7 in human colorectal cancer. Am J Pathol 155(4): 1033–1038 10514384 10.1016/s0002-9440(10)65204-2 PMC1867011

[bib6] Brennan KR, Brown AM (2004) Wnt proteins in mammary development and cancer. J Mammary Gland Biol Neoplasia 9(2): 119–131 15300008 10.1023/B:JOMG.0000037157.94207.33

[bib7] Coppola D, Szabo M, Boulware D, Muraca P, Alsarraj M, Chambers AF, Yeatman TJ (2004) Correlation of osteopontin protein expression and pathological stage across a wide variety of tumor histologies. Clin Cancer Res 10(1 Pt 1): 184–190 14734468 10.1158/1078-0432.ccr-1405-2

[bib8] Dawson PJ, Wolman SR, Tait L, Heppner GH, Miller FR (1996) MCF10AT: a model for the evolution of cancer from proliferative breast disease. Am J Pathol 148(1): 313–319 8546221 PMC1861604

[bib9] DiMeo TA, Anderson K, Phadke P, Fan C, Perou CM, Naber S, Kuperwasser C (2009) A novel lung metastasis signature links Wnt signaling with cancer cell self-renewal and epithelial-mesenchymal transition in basal-like breast cancer. Cancer Res 69(13): 5364–5373 19549913 10.1158/0008-5472.CAN-08-4135 PMC2782448

[bib10] El-Tanani M, Barraclough R, Wilkinson MC, Rudland PS (2001a) Metastasis-inducing DNA regulates the expression of the osteopontin gene by binding the transcription factor Tcf-4. Cancer Res 61(14): 5619–5629 11454716 10.1100/tsw.2002.238

[bib11] El-Tanani M, Fernig DG, Barraclough R, Green C, Rudland P (2001b) Differential modulation of transcriptional activity of estrogen receptors by direct protein-protein interactions with the T cell factor family of transcription factors. J Biol Chem 276(45): 41675–41682 11522780 10.1074/jbc.M103966200

[bib12] El-Tanani MK, Barraclough R, Wilkinson MC, Rudland PS (2001c) Regulatory region of metastasis-inducing DNA is the binding site for T cell factor-4. Oncogene 20(14): 1793–1797 11313926 10.1038/sj.onc.1204358

[bib13] Farago M, Dominguez I, Landesman-Bollag E, Xu X, Rosner A, Cardiff RD, Seldin DC (2005) Kinase-inactive glycogen synthase kinase 3beta promotes Wnt signaling and mammary tumorigenesis. Cancer Res 65(13): 5792–5801 15994955 10.1158/0008-5472.CAN-05-1021

[bib14] Graham TA, Ferkey DM, Mao F, Kimelman D, Xu W (2001) Tcf4 can specifically recognize beta-catenin using alternative conformations. Nat Struct Biol 8(12): 1048–1052 11713475 10.1038/nsb718

[bib15] Hoverter NP, Waterman ML (2008) A Wnt-fall for gene regulation: repression. Sci Signal 1(39): pe43 18827220 10.1126/scisignal.139pe43

[bib16] Khramtsov AI, Khramtsova GF, Tretiakova M, Huo D, Olopade OI, Goss KH (2010) Wnt/beta-catenin pathway activation is enriched in basal-like breast cancers and predicts poor outcome. Am J Pathol 176(6): 2911–2920 20395444 10.2353/ajpath.2010.091125 PMC2877852

[bib17] Mann B, Gelos M, Siedow A, Hanski ML, Gratchev A, Ilyas M, Bodmer WF, Moyer MP, Riecken EO, Buhr HJ, Hanski C (1999) Target genes of beta-catenin-T cell-factor/lymphoid-enhancer-factor signaling in human colorectal carcinomas. Proc Natl Acad Sci USA 96(4): 1603–1608 9990071 10.1073/pnas.96.4.1603 PMC15532

[bib18] Matsuda Y, Schlange T, Oakeley EJ, Boulay A, Hynes NE (2009) WNT signaling enhances breast cancer cell motility and blockade of the WNT pathway by sFRP1 suppresses MDA-MB-231 xenograft growth. Breast Cancer Res 11(3): R32 19473496 10.1186/bcr2317 PMC2716500

[bib19] McAllister SS, Gifford AM, Greiner AL, Kelleher SP, Saelzler MP, Ince TA, Reinhardt F, Harris LN, Hylander BL, Repasky EA, Weinberg RA (2008) Systemic endocrine instigation of indolent tumor growth requires osteopontin. Cell 133(6): 994–1005 18555776 10.1016/j.cell.2008.04.045 PMC4121664

[bib20] Mestdagt M, Polette M, Buttice G, Noel A, Ueda A, Foidart JM, Gilles C (2006) Transactivation of MCP-1/CCL2 by beta-catenin/TCF-4 in human breast cancer cells. Int J Cancer 118(1): 35–42 16003740 10.1002/ijc.21291 PMC2965755

[bib21] Michaelson JS, Leder P (2001) Beta-catenin is a downstream effector of Wnt-mediated tumorigenesis in the mammary gland. Oncogene 20(37): 5093–5099 11526497 10.1038/sj.onc.1204586

[bib22] Miller FR, Soule HD, Tait L, Pauley RJ, Wolman SR, Dawson PJ, Heppner GH (1993) Xenograft model of progressive human proliferative breast disease. J Natl Cancer Inst 85(21): 1725–1732 8411256 10.1093/jnci/85.21.1725

[bib23] Pannequin J, Delaunay N, Buchert M, Surrel F, Bourgaux JF, Ryan J, Boireau S, Coelho J, Pelegrin A, Singh P, Shulkes A, Yim M, Baldwin GS, Pignodel C, Lambeau G, Jay P, Joubert D, Hollande F (2007) Beta-catenin/Tcf-4 inhibition after progastrin targeting reduces growth and drives differentiation of intestinal tumors. Gastroenterology 133(5): 1554–1568 17920061 10.1053/j.gastro.2007.08.023

[bib24] Quandt K, Frech K, Karas H, Wingender E, Werner T (1995) MatInd and MatInspector: new fast and versatile tools for detection of consensus matches in nucleotide sequence data. Nucleic Acids Res 23(23): 4878–4884 8532532 10.1093/nar/23.23.4878 PMC307478

[bib25] Roose J, Huls G, van Beest M, Moerer P, van der Horn K, Goldschmeding R, Logtenberg T, Clevers H (1999) Synergy between tumor suppressor APC and the beta-catenin-Tcf4 target Tcf1. Science 285(5435): 1923–1926 10489374 10.1126/science.285.5435.1923

[bib26] Rudland PS, Platt-Higgins A, El-Tanani M, De Silva Rudland S, Barraclough R, Winstanley JH, Howitt R, West CR (2002) Prognostic significance of the metastasis-associated protein osteopontin in human breast cancer. Cancer Res 62(12): 3417–3427 12067984

[bib27] Shi Z, Hodges VM, Dunlop EA, Percy MJ, Maxwell AP, El-Tanani M, Lappin TR (2010) Erythropoietin-induced activation of the JAK2/STAT5, PI3K/Akt, and Ras/ERK pathways promotes malignant cell behavior in a modified breast cancer cell line. Mol Cancer Res 8(4): 615–626 20353997 10.1158/1541-7786.MCR-09-0264

[bib28] Shulewitz M, Soloviev I, Wu T, Koeppen H, Polakis P, Sakanaka C (2006) Repressor roles for TCF-4 and Sfrp1 in Wnt signaling in breast cancer. Oncogene 25(31): 4361–4369 16532032 10.1038/sj.onc.1209470

[bib29] Smalley MJ, Dale TC (1999) Wnt signalling in mammalian development and cancer. Cancer Metastasis Rev 18(2): 215–230 10728985 10.1023/a:1006369223282

[bib30] Stambolic V, Ruel L, Woodgett JR (1996) Lithium inhibits glycogen synthase kinase-3 activity and mimics wingless signalling in intact cells. Curr Biol 6(12): 1664–1668 8994831 10.1016/s0960-9822(02)70790-2

[bib31] Suzuki H, Toyota M, Carraway H, Gabrielson E, Ohmura T, Fujikane T, Nishikawa N, Sogabe Y, Nojima M, Sonoda T, Mori M, Hirata K, Imai K, Shinomura Y, Baylin SB, Tokino T (2008) Frequent epigenetic inactivation of Wnt antagonist genes in breast cancer. Br J Cancer 98(6): 1147–1156 18283316 10.1038/sj.bjc.6604259 PMC2275475

[bib32] Takahashi M, Tsunoda T, Seiki M, Nakamura Y, Furukawa Y (2002) Identification of membrane-type matrix metalloproteinase-1 as a target of the beta-catenin/Tcf4 complex in human colorectal cancers. Oncogene 21(38): 5861–5867 12185585 10.1038/sj.onc.1205755

[bib33] van de Wetering M, Sancho E, Verweij C, de Lau W, Oving I, Hurlstone A, van der Horn K, Batlle E, Coudreuse D, Haramis AP, Tjon-Pon-Fong M, Moerer P, van den Born M, Soete G, Pals S, Eilers M, Medema R, Clevers H (2002) The beta-catenin/TCF-4 complex imposes a crypt progenitor phenotype on colorectal cancer cells. Cell 111(2): 241–250 12408868 10.1016/s0092-8674(02)01014-0

[bib34] Veeck J, Niederacher D, An H, Klopocki E, Wiesmann F, Betz B, Galm O, Camara O, Durst M, Kristiansen G, Huszka C, Knuchel R, Dahl E (2006) Aberrant methylation of the Wnt antagonist SFRP1 in breast cancer is associated with unfavourable prognosis. Oncogene 25(24): 3479–3488 16449975 10.1038/sj.onc.1209386

[bib35] Wissmann C, Wild PJ, Kaiser S, Roepcke S, Stoehr R, Woenckhaus M, Kristiansen G, Hsieh JC, Hofstaedter F, Hartmann A, Knuechel R, Rosenthal A, Pilarsky C (2003) WIF1, a component of the Wnt pathway, is down-regulated in prostate, breast, lung, and bladder cancer. J Pathol 201(2): 204–212 14517837 10.1002/path.1449

[bib36] Woodward WA, Chen MS, Behbod F, Alfaro MP, Buchholz TA, Rosen JM (2007) WNT/beta-catenin mediates radiation resistance of mouse mammary progenitor cells. Proc Natl Acad Sci USA 104(2): 618–623 17202265 10.1073/pnas.0606599104 PMC1766434

[bib37] Yook JI, Li XY, Ota I, Hu C, Kim HS, Kim NH, Cha SY, Ryu JK, Choi YJ, Kim J, Fearon ER, Weiss SJ (2006) A Wnt-Axin2-GSK3beta cascade regulates Snail1 activity in breast cancer cells. Nat Cell Biol 8(12): 1398–1406 17072303 10.1038/ncb1508

[bib38] Yuen HF, Chan YF, Grills C, McCrudden CM, Gunasekharan V, Shi Z, Wong AS, Lappin TR, Chan KW, Fennell DA, Khoo US, Johnston PG, El-Tanani M (2011) Polyomavirus enhancer activator 3 protein promotes breast cancer metastatic progression through Snail-induced epithelial-mesenchymal transition. J Pathol 224(1): 78–89 21404275 10.1002/path.2859

[bib39] Yuen HF, Kwok WK, Chan KK, Chua CW, Chan YP, Chu YY, Wong YC, Wang X, Chan KW (2008) TWIST modulates prostate cancer cell-mediated bone cell activity and is upregulated by osteogenic induction. Carcinogenesis 29(8): 1509–1518 18453541 10.1093/carcin/bgn105

[bib40] Zhang J, Li Y, Liu Q, Lu W, Bu G (2010) Wnt signaling activation and mammary gland hyperplasia in MMTV-LRP6 transgenic mice: implication for breast cancer tumorigenesis. Oncogene 29(4): 539–549 19881541 10.1038/onc.2009.339 PMC2813429

